# Developing a shared decision-making intervention to support choices about prophylactic cranial irradiation treatment for small-cell lung cancer: a study protocol

**DOI:** 10.1136/bmjopen-2025-114379

**Published:** 2026-10-14

**Authors:** Katie Dixon, Marianne Aznar, Hilary Bekker, Tracey Olivia Burke, Clare Dempsey, Emma Halkyard, Ashley Horne, Corinne Faivre-Finn

**Affiliations:** 1 Division of Cancer Sciences, The University of Manchester Faculty of Biology, Medicine and Health, Manchester, UK; 2 Leeds Institute of Health Sciences, University of Leeds, Leeds, UK; 3 Patient Representative, Buxton, UK; 4 Department of Thoracic Oncology, The Christie NHS Foundation Trust, Manchester, UK

**Keywords:** QUALITATIVE RESEARCH, Patient-Centered Care, Decision Making, Clinical Protocols, Lung Neoplasms, Radiation oncology

## Abstract

**Abstract:**

**Introduction:**

Prophylactic cranial irradiation (PCI) is part of the standard of care for small cell lung cancer (SCLC) aiming to reduce the risk of brain metastases. However, PCI remains controversial due to its potential neurocognitive side effects, which can impact the quality of life, particularly among older and frailer patients. These trade-offs make the decision highly preference-sensitive, requiring careful consideration of individual patient values, goals and expectations. Shared decision-making (SDM) offers a structured approach to supporting informed and values-based choices between patients and healthcare professionals. Despite its importance, SDM is not routinely integrated into PCI consultations and patients often report a lack of support in weighing the benefits and risks. This protocol outlines research to develop a complex SDM intervention to support patients with SCLC and health professionals in making this PCI decision together more effectively.

**Methods and analysis:**

This mixed-methods study is guided by the Medical Research Council framework for developing and evaluating complex interventions. The study will be conducted in three phases at The Christie National Health Service Foundation Trust. Phase 1 involves an environmental scan of UK-based patient information resources and clinical guidelines to evaluate the availability, quality and consistency of existing SDM resources for PCI. Phase 2 uses qualitative and quantitative methods including audio-recorded consultations, questionnaires, and interviews with patients, companions and healthcare professionals. This will explore current decision-making practices, barriers, facilitators and support needs. Data from phase 1 and 2 will be triangulated to inform the design of an SDM intervention prototype. Phase 3 includes a user-centred, iterative design process involving feedback from patient and public involvement groups to develop and assess the acceptability and contextual fit of the prototype intervention.

**Ethics and dissemination:**

Ethical approval has been granted by the North West – Greater Manchester Central Research Ethics Committee (25/NW/0160) for phase 2. The outputs from this study will be shared through Figshare, publications, conferences and the researcher’s PhD thesis.

**Strengths and limitations of this study**

- A mixed-methods study design developed with a multidisciplinary steering group follows published frameworks to explore current information, practice and stakeholder perceptions which will inform the development of a decision support intervention.
- Patient and public involvement advisory groups will advise on the development of the decision support intervention.
- The study is limited to English resources and fluent English-speaking participants. In addition, phase 2 of the study, which includes interviews with patients, companions and healthcare professionals, is single-centre.
- The limitations will reduce the transferability of the findings to other languages or underserved populations.

## Introduction

 Small cell lung cancer (SCLC) is an aggressive subtype of lung cancer,^1^ representing approximately 12%–15% of all cases.^2^ SCLC is classified into two categories: limited-stage (LS) disease and extensive stage (ES). LS disease is where the tumour is confined to the thorax and can be encompassed within a single radiation port. ES disease includes all cases that do not meet these criteria. SCLC is characterised by rapid progression and early development of treatment resistance, despite its initial sensitivity to chemotherapy and radiotherapy. Although therapeutic advances have been made, the overall prognosis remains poor.^3^

A major clinical concern in SCLC is the high risk of developing brain metastases.^4^ To address this, prophylactic cranial irradiation (PCI) has been used to target potential microscopic disease within the brain.^5^ Randomised controlled trials and meta-analyses have demonstrated that PCI can reduce the incidence of brain metastases by approximately 50% and improve overall survival, particularly in the limited stage setting.^6 7^ PCI is routinely considered for patients with LS-SCLC following chemoradiotherapy.^5^ PCI is also considered for those with ES-SCLC who respond to systemic treatment, typically chemotherapy and immunotherapy, with chest radiotherapy reserved for selected patients.^8^ However, concerns about the long-term adverse effects of PCI, an evolving treatment landscape and conflicting trial results have led to increasing scrutiny of its role in contemporary practice.^7^

Neurocognitive decline is a well-recognised long-term effect of PCI, particularly among patients who are older or those with comorbidities.^9 10^ Additionally, other side effects such as prolonged fatigue, nausea, headaches and alopecia may further reduce the quality of life.^11^ These considerations are increasingly important as survival improves and more patients live with the long-term effects of treatment.

The evidence base for PCI has also become more complex. A more recent randomised study in ES-SCLC found no survival benefit when comparing PCI to brain MRI surveillance, leading to the adoption of MRI surveillance as an alternative.^12^ However, much of the evidence supporting PCI originates from trials conducted before the era of MRI surveillance^13^ or the incorporation of immunotherapy into the treatment paradigm for both LS and ES disease.^14 15^

Differences between earlier and contemporary studies may reflect changes in staging, imaging availability, surveillance practices and systematic therapies.^13^ Consequently, clinicians and patients must make decisions within a context of evolving and conflicting evidence landscape.^16^ In addition, despite these developments, MRI surveillance is not widely implemented as standard of care in the UK due to limited evidence, guideline ambiguity and resource constraints.^13 17^ As a result, many patients are effectively deciding between PCI and no PCI rather than between PCI and MRI surveillance programmes.^16^ This further highlights the complexity of PCI decision-making and the importance of shared decision-making (SDM).^18^

Guidelines have struggled to keep pace with this evolving evidence base. Consequently, the European Society for Medical Oncology (ESMO)^1^ has recognised the need for SDM to support patient-centred discussions around PCI. Nevertheless, the clinical pathway remains unclear.^8^ The lack of consensus and rapid evolution in evidence contribute to the variations in practice and divergent views. This could introduce uncertainty and the potential for bias during clinical consultations.^18 19^

Patients with SCLC therefore face a difficult decision: to proceed with PCI, to decline it or, where available, to opt for MRI surveillance. This decision requires careful weighing of the possible survival benefit against the risk of neurocognitive effects and impact on quality of life.^9^ However, evidence suggests that SDM practices are inconsistently applied in this context.^18 19^ Many patients have reported receiving insufficient information and feel inadequately involved in the decision-making process to support their deliberation around PCI.^18^ There are SDM tools such as patient decision aids (PtDA), structured consultation and clinician training that have shown potential in supporting more informed and value-based decisions. Yet, few resources are currently tailored for PCI discussions in SCLC.^18–20^

Given the persistent uncertainties in the evidence, the variation in clinical practice and the absence of decision-support resources, there is a clear need for an evidence-informed SDM intervention tailored to the modern management of SCLC. This study addresses that gap by systematically identifying patient-facing resources, exploring the perspectives of key stakeholders and co-designing a prototype SDM intervention tailored to the UK oncology setting. In doing so, it aims to support informed, collaborative decision-making for patients with SCLC facing the complex and preference-sensitive decision about PCI.

## Methods and analysis

A one-arm convergent mixed-methods study is intended to design and explore the acceptability of a PCI SDM intervention for patients living with SCLC and health professionals.

The protocol follows the GUIDance for the rEporting of intervention Development (GUIDED)^21^. Specific components are reported according to relevant methodological frameworks: Preferred Reporting Items for Systematic reviews and Meta-Analyses extension for Scoping Reviews (PRISMA-ScR)^22^ for the environmental scan, the Good Reporting of a Mixed Methods Study (GRAMMS)^23^ guidelines and the Template for Intervention Description and Replication (TiDieR) checklist and guide^24^ for describing the intervention.

The research method is informed by established frameworks for intervention development. The Medical Research Council (MRC) framework for developing and evaluating complex interventions^25 26^ will provide overarching guidance. Conceptual direction for designing interventions to support multiple decision-makers in making decisions well is provided by the Making Informed Decisions Individually and Together (MIND-IT) in the healthcare framework.^27^ The Ottawa Decision Support Framework (ODSF)^28^ and International Patient Decision Aid Standards (IPDAS)^29^ provide specific guidance for decision support interventions and methods guiding a user-centred approach to intervention development.^30 31^

A steering group comprising patients with lived experiences of SCLC, clinical oncologists, medical physicists, lung cancer nurse specialists and medical decision specialists are involved in shaping the study design, reviewing participant-facing materials and co-developing the decision support intervention. They will also contribute to interpreting findings and plan dissemination. In subsequent publications, involvement will be reported using the Guidance for Reporting Involvement of Patients and the Public (GRIPP2)^32^ short form checklist to ensure transparent and meaningful reporting of patient and public involvement (PPI) activities and their impact.

### Context

The research will be conducted at The Christie National Health Service (NHS) Foundation Trust, a specialised tertiary cancer centre.

Following initial treatment for SCLC, patients are referred to clinical/radiation oncologists to discuss the option of PCI. At this stage, patients are typically offered two options:

1. Proceed with PCI, which remains part of the standard of care.
2. Decline PCI and continue with the remainder of standard of care.

In the UK, brain MRI surveillance is not currently a standard NHS option recommended by national guidelines.

### Overview of the study and objectives

1. Phase 1: an environmental scan^33 34^ will be conducted to collate existing resources used to support patient and healthcare professional decision-making regarding PCI treatment for SCLC within UK services. Quality, completeness and decision-support content of these resources will be evaluated (figure 1).
2. Phase 2: explore how patients and healthcare professionals make decisions regarding PCI within current SCLC treatment pathways using: semi-structured interview methods with patients and companions to explore barriers and facilitators related to SDM; observation methods using audio-recordings of PCI consultations to observe real-time conversations and analyse how treatment options are presented and discussed; and questionnaire methods evaluating patient and healthcare professional experiences and perceived ability to implement SDM practices.
3. Phase 3: an iterative, user-centred design process to develop a prototype SDM intervention to enable clinicians to support patients and make informed decisions about PCI in the context of their cancer care and daily life. Acceptability of the relevance, clarity and usability of the intervention will be explored.

**Figure 1:**
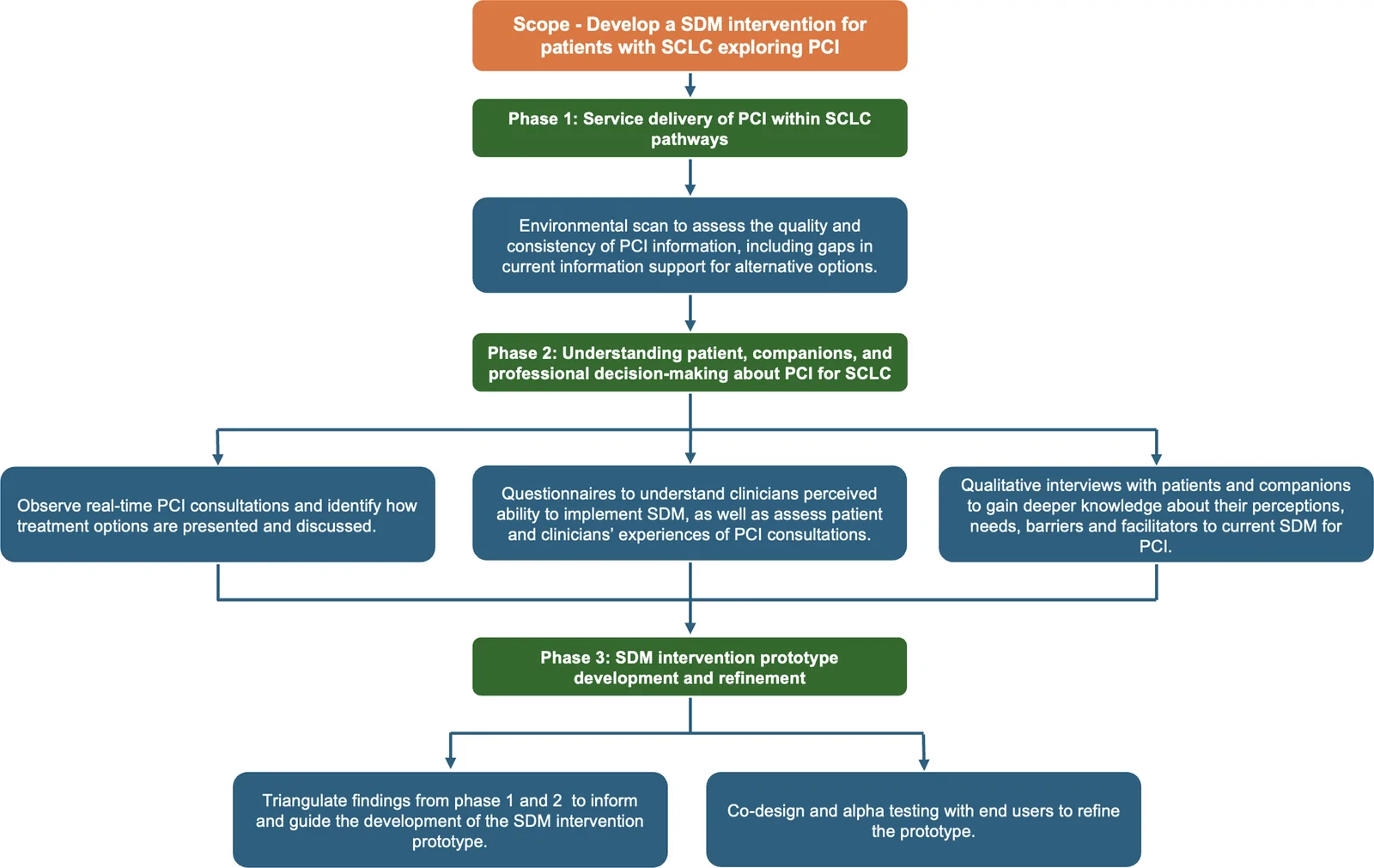
An outline of the SDM intervention development process for PCI discussions in the SCLC treatment pathway. PCI, prophylactic cranial irradiation; SCLC, small cell lung cancer; SDM, shared-decision making.

### Phase 1: service delivery of PCI within SCLC pathways

#### Purpose

Environmental scans^33^ provide a systematic method for gathering and organising information from diverse sources to map and evaluate the current landscape of services, resources, policies and practices. They have a broader scope than systematic reviews and can uncover resources that may not have undergone a published evaluation.^33 34^ In phase 1, the environmental scan will examine PCI information resources to identify gaps in the availability, quality, consistency and decision-support content of existing resources.^33 34^ This will inform the broader needs assessment and guide the development of a PCI decision-support intervention.

#### Sources

Patient-facing PCI information resources provided by UK radiotherapy centres, current publicly available UK oncology charity websites (eg, Cancer Research UK, Macmillan and Roy Castle), and relevant international clinical guidelines will be included for review.

Resources from non-UK radiotherapy centres or non-UK oncology charity websites will be excluded.

#### Method

To access the patient-facing information provided by radiotherapy centres, a one-off anonymous national survey will be distributed to radiotherapy centres across the UK. The survey will be distributed to publicly available contacts for these centres or through professional contacts. The survey requests details about the information they provide to patients regarding PCI and its alternatives. The centres will have the option to submit written information materials alongside the survey for review by the research team. They will also be invited to describe any verbal information routinely provided in place of written materials.

This phase is currently ongoing.

#### Data analysis

The leading researcher will conduct qualitative content analysis of patient-facing information resources. The quality and completeness of each resource will be assessed using the IPDAS checklist,^29^ with resources scored accordingly. To limit bias, a second member of the research team will review the resources using the IPDAS checklist.

Quantitative data will be managed in IBM SPSS (V.30) and summarised through descriptive statistics. Given the exploratory nature of this environmental scan, no inferential or hypothesis-testing statistical analyses will be performed. The collected data will be stored securely on an encrypted server and accessible only to authorised research team members.

### Phase 2: patient, companion and professional decision-making about PCI for SCLC

#### Purpose

This phase uses a mix of methods, including audio-recording of consultations, questionnaires and interviews to identify the experience and needs of stakeholders (healthcare professionals, patients and companions) to engage in SDM about PCI.

#### Participants and inclusion/exclusion criteria

Patients with SCLC: adults (≥18 years) with a confirmed diagnosis of stage I–IV SCLC, who are currently being offered or have recently considered PCI, will be eligible. Participants must be able to provide informed consent and participate in English-language interviews. Recruitment will be limited to patients treated at The Christie NHS Foundation Trust. Patients with cognitive impairments that preclude informed consent will be excluded.

Companions: adults (≥18 years) who have supported a patient with SCLC through the decision of whether to have PCI or to have no treatment. Companions include friends, family members and carers if they speak and understand English.

Healthcare professionals working in SCLC: medical or clinical oncologists involved in SCLC care at The Christie NHS Foundation Trust and who currently offer or discuss PCI as a treatment option will be invited to participate. Oncologists not working with SCLC and PCI will be excluded.

#### Researcher description

Participant recruitment, data collection and analysis will be conducted by a PhD researcher with a background in health psychology. Their professional training and experience have included extensive qualitative and quantitative research methods. Reflective notes will be maintained throughout the study to consider how assumptions and prior knowledge may influence data collection, analysis and interpretation.

#### Recruitment and consent

Participants will be recruited through purposive, criterion-based sampling^35^ based on inclusion criteria above. However, due to practical constraints and project timelines, a convenience recruitment strategy will also be applied by sourcing all participants from a single tertiary care centre.^36^ Recruitment opened in October 2025 and is currently ongoing. Data collection is expected to continue until September 2026.

##### 2a: audio-recording of PCI consultations

Healthcare professional participants will be screened against the eligibility criteria by the thoracic oncologist within the research team. Following this, the researcher will approach the healthcare professionals. They will be recruited first and consented prior to patient recruitment. Participating healthcare professionals will review their clinic list and will identify patients who meet the eligible criteria. On the consultation day, the healthcare professional will introduce the researcher to the patient. The researcher will explain the study and obtain written informed consent from the patient and any accompanying individuals.

##### 2b: questionnaires

All participants involved in the audio-recorded consultations will be invited to complete the questionnaires, with consent already obtained.

##### 2c: qualitative interviews

Patients will be identified and recruited in the same way as the consultation recording group. Interested individuals and accompanying companions will be offered information about the interviews. They will be asked to sign a ‘consent to contact’ form for the researcher to contact them for the interview. Verbal informed consent will be obtained immediately prior to the interviews.

### Data collection methods

#### 2a: audio-recording of PCI consultations

Consultations will be audio-recorded to analyse how management options are communicated and discussed, and to identify areas where additional decision support could be beneficial. The consultations will be recorded in full using encrypted recording devices unless otherwise specified. The researcher will be positioned in the consultation room in a way which limits interruption to the consultation and reduces clinical pressure on participating oncologists by ensuring the recording runs smoothly. The encrypted recording device will be placed nearby in an open space to enable clear audio detection.^37^ Each consultation is expected to last approximately 20 min.^38^

The researcher will generate an automatic transcript using Microsoft Word and will then review them word by word while listening to the original audio twice. This process will help identify transcription errors, verify precise speaker attributions and incorporate relevant non-verbal cues (eg, pauses and hesitations). This approach ensures accuracy while preserving participants’ authentic voices.^37^ Transcriptions will be stored securely on encrypted servers with access restricted to authorised personnel.

#### 2b: questionnaires

Questionnaires will be administered to both patients and healthcare professionals who participate in the audio-recorded consultations.

Before the consultations, the healthcare professionals will complete a one-off IcanSDM^39^ questionnaire, a validated 8-item questionnaire to measure the perceived ability of healthcare professionals to implement and practice SDM. Two additional statements will be included to assess their perception of SDM training: (1) *‘*I feel confident in applying shared decision-making principles in my clinical practice based on my training’ and (2) ‘I feel I would benefit from additional training to enhance my ability to support patients in making informed decisions’. Statements from the IcanSDM^39^ and the training questions will be rated from 0 (strongly disagree) to 10 (strongly agree).

Following the consultations, patients will be invited to complete the patient-facing Experience of Shared Decision-Making (SHARED)^40^ questionnaire. The patient-facing SHARED^40^ questionnaire is a 10-item questionnaire developed using service improvement methods^41^ that assesses patient experience of making shared decisions with healthcare professionals about their care. In addition, healthcare professionals will complete the healthcare professional (HCP) SHARED questionnaire (online supplemental appendix 1), an 11-item plus an open-ended text box questionnaire adapted from the patient-facing SHARED.^40^ It assesses healthcare professionals’ experience of making shared decisions with patients about their care. The patient-facing SHARED^40^ and HCP-SHARED questionnaires are rated on a 5-point rating scale (disagree strongly to agree strongly). Healthcare professional questionnaires will be distributed via a secure Qualtrics link and patient’s questionnaires will be provided with a paper version to complete before leaving the clinic.

#### 2c: qualitative interviews

Individual semi-structured interviews will be conducted with patients and companions. These interviews aim to gain an in-depth understanding of the factors influencing PCI decision-making, including perceived barriers, facilitators and support needs. Interviews will run either face to face, by telephone or via Microsoft Teams according to participant preference, and audio-recorded using encrypted devices and secure platforms. An interview guide (online supplemental appendix 2) was developed using relevant decision-making frameworks^28 29 42^ to ensure coverage of topics related to study objectives. Keeping in mind the patient’s well-being and physical health, each interview is expected to last for approximately 30 min.

### Sample size

A total of 14–44 participants will be recruited across all cohorts.

For the consultations, this includes between 2–5 clinician participants who will agree to have 3–4 consultations recorded. Of these participants, 2–5 healthcare professionals will complete the IcanSDM^39^ questionnaires, 6–20 patients will complete the SHARED^40^ questionnaire and 6–20 healthcare professionals will complete the SHARED questionnaire.

There will be 8–24 participants in the patient and companion interviews.

This recruitment target aligns with qualitative research recommendations for sample size.^37^ Recruitment will continue until data saturation is reached, meaning no new themes or concepts emerge. If saturation occurs, data collection will be paused accordingly. This method will help to account for potential participant dropout, particularly among patients who are unable to complete interviews. A reflective log^43^ will be maintained throughout data collection to ensure transparency and rigour.^37^

No formal sample size calculation was performed for the questionnaires, as questionnaire data will be triangulated with qualitative findings from interviews and consultation recordings.

### Data analysis

All statistical analyses will be performed using IBM SPSS (V.30), while qualitative data will be managed and analysed using QSR Interventional NVivo (V.15) and Microsoft Word.

Qualitative data, including transcripts from the interviews and consultation recordings, will undergo thematic analysis to explore participant perceptions, experiences and beliefs of the participants.^37^ This allows the identification and examination of patterns and themes within data. The researcher will iteratively code the transcripts, using constant comparison to develop and refine emerging codes and themes.^37^

Given the anticipated small sample sizes, analyses will be exploratory and interpreted cautiously. Quantitative data from the SHARED^40 44^ and IcanSDM^39^ questionnaires, including two additional SDM training questions, will be analysed using descriptive statistics. Paired group comparisons between patient and healthcare professional responses will be performed using Wilcoxon signed-rank test. Unpaired comparisons will be performed using Mann-Whitney U tests and categorical variables will be analysed using Fisher’s exact test. Relationships between questionnaire scores will be explored through Spearman’s rank correlation coefficients. If the sample sizes are too small, these will be limited to descriptive statistics. Emphasis will be placed on effect sizes, CIs where appropriate, and patterns within the data rather than statistical significance alone.

Separately, the Decision Support Analysis Tool (DSAT-10)^45^ will be applied to consultation recordings to assess the current use of decision support as well as communication skills during a clinical encounter which aligns with the original intended use of the DSAT-10.^45 46^ The researcher will listen to each encounter twice and items will be marked according to whether these elements were adequately addressed during the consultation. Examples from the dialogue will be provided to support that the criterion was met. Although the researcher has had no previous experience using this tool, they familiarised themselves with the scoring framework and supporting literature before the analysis.^45 46^ These results will be discussed with the steering committee and included in the triangulation with the quantitative and qualitative results to enhance validity.

### Phase 3: development and refinement of the SDM intervention in the healthcare pathway

The SDM intervention aims to enable patients to evaluate all management options in relation to their potential expectations and values. This will facilitate informed trade-offs during discussions with SCLC specialists.

The development of the SDM intervention will be guided by established frameworks. This includes the IPDAS checklist^29^ and ODSF^28^ to ensure the essential criteria for a ‘good decision aid’ are met. Additionally, the National Institute for Health and Care Excellence guidelines for SDM^47^ and the MRC framework for complex interventions^25^ to guide the content and development of the intervention will be used. Furthermore, the MIND-IT in the healthcare framework^27^ will be used to involve stakeholders in the SDM intervention. These frameworks will ensure the intervention supports evidence-based, value-sensitive decisions aligned with best oncology practices.

The content and format of the initial intervention prototype will be informed by data collected in phases 1 and 2.^48^ The prototype will undergo co-design and alpha testing with patients, companions and healthcare professionals.^30 31 48^ This phase will focus on addressing the acceptability, usability, comprehensibility and potential integration of the intervention into routine clinical practice. Feedback obtained will refine and optimise the prototype prior to future feasibility and evaluation studies.^30 31 48^ Activities will include the following:

#### 3a patient and companions

Five to eight patients with SCLC and associated companions will be invited to review the prototype and participate in a semi-structured interview. Participants will be encouraged to comment on comprehensibility, acceptability, usefulness and suggest modifications.

#### 3b: healthcare professionals

Ten to fifteen healthcare professionals involved in the care of patients with SCLC, including clinical medical oncologists and specialist nurses, will be invited to review the prototype and complete a structured feedback survey. The survey will explore similar topics to patients and companions as well as exploring feasibility of implementation and space to suggest modifications.

Feedback will be reviewed by the steering group to support further refinement of the intervention.^30 31 48^ A modification log will be maintained to document changes made during the process. The iterative refinement process will result in an optimised prototype complex intervention.

##### Decision intervention output

Based on comparable oncology^49–53^ and current SDM guidelines,^25 28 29^ the final intervention may comprise multiple tools targeting all stakeholders (table 1). This includes:

PtDA: this will clearly describe the decision the patient is facing, provide an overview of the risk of brain metastases and outline the available options (ie, PCI and no PCI). It will present the advantages, disadvantages and consequences of each choice, using numerical probabilities.^31 54 55^ Interactive elements and visuals will engage patients, guiding them through the decision process, encouraging communication with healthcare professionals and companions, and helping to clarify personal values and preferences.^56^ The PtDA is intended to be provided before or during the patients’ initial consultation about PCI, depending on the results from phases 1 and 2. This will also include guidance on its use and optional access from home for further reflection.

**Table 1:** Anticipated components of the SDM intervention

Component	Purpose	Format
PtDA	Support informed decision-making and encourage SDM between stakeholders	Leaflet, digital, video, printable tool
Health professional guide for the PtDA and SDM conversation	Ensure the PtDA is used effectively and consistently	Leaflet, digital, printable tool
Health professional training	Facilitate SDM into practice	Workshop or e-learning

PtDA, patient decision aids; SDM, shared decision-making.

Healthcare professionals guide: this guide will raise awareness of the PtDA purposes and application, providing practical guidance on structuring PCI discussions to minimise bias, clearly present options and evidence and incorporate patient preferences effectively.^57 58^ Informed by current guidance on decision coaching.

SDM training for healthcare professionals: training will be informed by current SDM intervention training programmes^59–61^ with a combination of brief online sessions and, if appropriate, problem-solving and feedback meetings to discuss SDM in practice. Content may include differentiating patient-centred care from SDM, case scenarios, PtDA use and post-training assessments.^47 62 63^

### Outcomes and outputs

#### Phase 1: service delivery of PCI within SCLC pathways

- Characterisation of existing PCI information resources.
- Identification of gaps, inconsistencies and variations in current informational support provided to patients regarding PCI within the SCLC treatment pathway.

#### Phase 2a, 2b and 2c: patient, companion and professional decision-making about PCI for SCLC

- Understanding of current decision-making behaviours.
- Identification of barriers, facilitators and support needs for all stakeholders necessary to make informed decisions about PCI.

#### Phase 3a and 3b: user-centred design of SDM support intervention

- A prototype SDM intervention developed using the comprehensive data gathered from Phases 1 and 2 and alpha testing through co-design activities.

### Patient and public involvement

PPI will be continuously incorporated throughout the study. To date, one representative who has lived experience of SCLC and PCI has been involved as a steering group member and has been involved in shaping the study design and reviewing participant-facing materials. During study development, our patient representative reviewed the protocol, patient-facing information sheets, questionnaires and interview questions. We completed two sessions to ensure all topics were covered. Their feedback led to revisions that improved clarity, sensitivity and relevance of the research. It also highlighted the importance of clear, accessible patient information regarding PCI and has helped align the project with issues most relevant to those making PCI decisions. Additional PPI groups will be incorporated to support creating dissemination plans.

## Discussion

Healthcare professionals and patients with SCLC are confronted with a challenging decision regarding PCI or no PCI to manage the risk of brain metastases.^13 18^ These preference-sensitive decisions involve trade-offs between uncertain survival benefits and potential neurocognitive risks, which could impact quality of life. This necessitates personalised, value-sensitive deliberation between patients and healthcare professionals.^13 18^ Yet, despite the clear rationale for SDM in this setting, tools to support such conversations are limited and rarely integrated into routine clinical practice.^18 19^ Therefore, this protocol outlines a multi-phase study aligned with complex intervention guidelines.^25 28^ The study develops a prototype SDM intervention tailored to PCI decisions within the SCLC treatment pathway. It aims to enhance the quality and consistency of decision-making around PCI by prompting unbiased, collaborative, and patient-centred care.^64–66^

A limitation of the study is the inclusion of a single tertiary centre and is limited to fluent English-speaking participants, potentially affecting transferability of findings. Due to the scope of the PhD project, the evaluation of implementation or effectiveness is not included. Therefore, further research will be required to assess the impact of the developed intervention in routine clinical practice.

Despite these limitations, this study offers new insights for decision support within the context of PCI in SCLC. Additionally, it expands decision-making literature by treating SDM as a multi-level process, exploring perspectives within and beyond the clinician-patient dyad.^27 67^ This will empower patients to make informed choices that reflect their values and preferences, improving their experience and sense of control in managing their cancer. If this work is successful, it will offer practical insights for embedding SDM more broadly across NHS oncology services and support the delivery of more responsive, equitable cancer care.

## Ethics and dissemination

### Research ethics approval

Ethical approval for Phases 2a, 2b, and 2c has been granted by the North West – Greater Manchester Central Research Ethics Committee, reference number 25/NW/0160.

### Access to data

All fully anonymised data generated or analysed during this research will be made publicly available in Figshare at the University of Manchester following publication.

### Dissemination policy

On study completion, results will be disseminated in line with the sponsor dissemination policy. A comprehensive final report will be submitted to the research ethics committee within 12 months of study closure. Findings will also be submitted for publication in peer-reviewed international journals, presentation at both national and international conferences and incorporated into the researcher’s PhD thesis.

## Supplementary material

10.1136/bmjopen-2025-114379 online supplemental file 1
