# Supplementary material for: Developing a shared decision-making intervention to support choices about prophylactic cranial irradiation treatment for small-cell lung cancer: a study protocol
Source: BMJ Open. 2026 Oct 14;16(10):e114379. doi: 10.1136/bmjopen-2025-114379 (PMC13691757; doi:10.1136/bmjopen-2025-114379)
Supplement: Supplementary file 1 [file bmjopen-16-10-s001.pdf]

Supplemental Material

Appendix 1: The HCP-SHARED Questionnaire

Professional experience of Shared Decision-Making (SHARED: professional-specific lung cancer).

We are interested in your views around your discussions with the patient diagnosed with small cell lung cancer when discussing risk of brain metastasis. We use the word option to mean the treatment choices you might talk about during these consultations. Options can be prophylactic cranial irradiation (PCI) or no PCI or an alternative treatment. Please answer all questions below as accurately as possible. There is opportunity to write any additional comments in a free text box at the end of the questionnaire.

For each statement below, tick the box that most closely matches how much you agree or disagree with it:

During a consultation…

	I talked about…	**Agree Strongly**	**Agree**	**Not sure**	**Disagree**	**Disagree Strongly**
**1.**	all relevant options available to my patient including the option not to have a procedure	☐	☐	☐	☐	☐
**2.**	whether or not there was a clinically best option for this health problem.	☐	☐	☐	☐	☐
**3.**	my views about the options.	☐	☐	☐	☐	☐
	I asked...	**Agree Strongly**	**Agree**	**Not sure**	**Disagree**	**Disagree Strongly**
**4.**	I asked the patient for their views about the options.	☐	☐	☐	☐	☐
**5.**	I asked family members or companions for their views about the decision	☐	☐	☐	☐	☐
	The patient…	**Agree Strongly**	**Agree**	**Not sure**	**Disagree**	**Disagree Strongly**
**6.**	talked about what is important to them about this decision.	☐	☐	☐	☐	☐
**7.**	talked about why one option suited them better than another.	☐	☐	☐	☐	☐

**8.**	talked about the risks and benefits of each option for their health and lifestyle.	☐	☐	☐	☐	☐

	I felt…	**Agree Strongly**	**Agree**	**Not sure**	**Disagree**	**Disagree Strongly**
**9.**	it would be appropriate for patient to choose any options we talked about.	☐	☐	☐	☐	☐
**10.**	confident the decision made aligned with my patient's values for this clinical situation.	☐	☐	☐	☐	☐
**11.**	the patient and I reached a shared understanding of the option that fits best with their needs and circumstances.	☐	☐	☐	☐	☐

Please use this box to write any comments regarding this questionnaire and the answers you have given. Do not to complete the free text box with any potentially identifiable information, such as names or places.

Appendix 2: Interview guide for both patients and companion interviews

DISCUS: Development and Evaluation of Decision Support Tools in Routine Oncology Practice Interview topic guide for patients and personal support.

Before the interview starts:

"Thank you for confirming consent and agreeing to be interviewed as part of the DISCUS study. I would like to start the interview now and this will be recorded to allow me to transcribe what is discussed. As a reminder this research aims to explore the decision-making process around prophylactic cranial irradiation offered to patients diagnosed with extensive stage and limited stage small cell lung cancer."

Topic	Key Questions	Prompts
**Background**	• Please describe to me what you think PCI/the PRIMALung trial is.	• Do you understand why these options were provided?
	• What is your understanding of risk and benefits/pros and cons when considering treatment?	
**The decision**	• Please tell me what choice you/the patient made following the consultation with the oncologist?	• Can you tell me more about the reasons for your choice?
	• Please tell me about your experiences of the consultation where the information about your choices was provided.	• How did you find the care and communication?<br>• Did you feel like you could ask questions in the consultation?<br>• Did you ask questions?<br>• Did you feel comfortable about asking questions after the consultation had finished for example, ringing up the nurse specialists?
**Information provided**	• Please could you discuss your thoughts on the information you/your relative was provided with during the consultation and the information provided to you to take home.	• What formats were the information provided in?<br>• Was an information booklet provided?<br>• How often did you refer to the information while you were at home?

		Did you access information on the internet/social media?<br>• Which format of information is your preference, considering information during the consultation and the takeaway information?<br>• How did it make you feel?
	• Please discuss your thoughts on how easy the information was to understand. Both in consultation and the information provided to take home.	• Was the information easy to understand/not understand?<br>• How did you feel about the amount of information provided?<br>• How did the information given to take away and read compare to the information given in the consultation?<br>• If you were given information about the PRIMALung study, what did you think about the content/length of the document?
	• Please tell me if there is anything that was missing in the consultation that you would have wanted.	• Would you change anything about the information provided?
**Important values**	• Please discuss what is most important in your day-to-day life when considering treatments.	• What are your goals and priorities when considering treatment?<br>• Does this differ to the longer-term goals you consider? If yes, how?
	• Tell me if and how these goals and values were considered during your decision?	• How did you take them into account when you were choosing the decision?<br>• Were they considered during the consultation? If yes, how? If no, why do you think they weren't?

	• Tell me if you think your goals and values might be different to what the clinicians, your family/friends, patients may consider?<br><br>• How important it is that goals and priorities are discussed.	• How might they differ?<br><br>• Why might they differ?
**Support**	• Tell me about the support when choosing treatment.<br><br>• Tell me if you think there could be other kinds of support provided to help with the decision process.	• Did you talk to others about the decision?<br><br>• Who did you talk to?<br><br>• Who did you find was the best support?<br><br>• If you received any conflicting opinions, how did you deal with this?
**Closing discussion**	• What do you think could or needs to change about the treatment and decision process.<br><br>• Is there anything else you would like to say before the interview finishes?	
