# Supplementary material for: Developing a shared decision-making intervention to support choices about prophylactic cranial irradiation treatment for small-cell lung cancer: a study protocol
Source: BMJ Open. 2026 Oct 14;16(10):e114379. doi: 10.1136/bmjopen-2025-114379 (PMC13691757; doi:10.1136/bmjopen-2025-114379)
Supplement: Supplementary file 2 [file bmjopen-2025-114379.reviewer_comments.pdf]

PEER REVIEW HISTORY

BMJ Open publishes all reviews undertaken for accepted manuscripts. Reviewers are asked to complete a checklist review form and are provided with free text boxes to elaborate on their assessment. These free text comments are reproduced below.

ARTICLE DETAILS

Title (Provisional)

Developing a Shared Decision-Making Intervention to Support Choices About Prophylactic Cranial Irradiation Treatment for Small-Cell Lung Cancer: A Study Protocol

Authors

Dixon, Katie; Aznar, Marianne; Bekker, Hilary; Burke, Tracey Olivia; Dempsey, Clare; Halkyard, Emma; Horne, Ashley; Faivre-Finn, Corinne

---

VERSION 1 - REVIEW

**Reviewer**	**1**
**Name**	**Butler, Jorie**
**Affiliation**	**University of Utah Health**
**Date**	**05-Jul-2026**
**COI**	**None**

This is a protocol paper focused on a study planned to assess the acceptability of shared decision making for small cell lung cancer treatment Prophylactic cranial irradiation (PCI). This is an important choice because there are risks for complications, particularly for older adults. Based on RCTs and meta-analyses – PCI is expected to reduce the likelihood of brain metastasis – though there are recent studies which show no benefit relative to MRI screening (which would not have the same risk). In the UK, MRI is not guideline concordant treatment although a European Oncology society has recognized the need for shared decision making conversations – these are not routinely happening.

The introduction clearly explains the need for the study. The complexity of the emerging evidence contrary to RCT and other evidence needs more explanation – it isn't clear if the authors are proposing a reason for the conflicting results (could the newer studies be more representative?) or if this is conceptualized as part of the complex landscape of a tough decision for patients and their clinicians. This is particularly important given the choices the patients will be offered (PCI or no PCI each choice accompanied by standard care which does NOT include MRI screening).

The study is a single arm convergent mixed-methods design. There are three phases 1) scan for environmental evidence 2) qualitative interviews, clinic visit observations, and clinician surveys about future SDM interventions and efficacy and 3) user-centered design of an SDM intervention.

Phase 1 methods. The environmental scan is focused on evaluation of patient-facing resources for IPDAS criteria. The analytic approach seems very reasonable but it could be argued that this is less of an environmental scan – given what may be a relatively narrow scope of materials – and more of a needs assessment focused on demonstrating the need for high quality decision aids. Although not a problem per se – it would enhance the rigor of the approach if the intention of the aim were harmonized more directly with the methods and analysis.

Phase 2 methods. In phase 2, the plan is to audiorecord consultations, collect questionnaire data, and perform semi-structured interviews. Inclusion and exclusion criteria for patients and healthcare professionals seem reasonable. The recruitment process is described as including "purposive sampling" but also employing convenience sampling. It should be made clear what the purposive sampling strategy is and how purposive sampling will be done. In addition, clarifying how convenience sampling strategies will be employed specifically in concert with purposive sampling (e.g., when purposive strategies fail? When there are insufficient numbers for purposive sampling?) should also be clarified.

The methods for data collection in phase 2 need further explication throughout. For example, in the consultation recordings section there should be more detail where will the recorder be placed in the room? What does preparing transcriptions "by the researcher" mean and how will quality be ensured?

It seems that healthcare providers complete two questionnaires, and patients only the patient facing SHARED version. Is this correct? Providing some additional information about each questionnaire, and more about the content for the supplemental provider questions would improve this section of the manuscript.

The qualitative interview description is quite vague. Who will complete the interviews? Have interview scripts been created? Is there a theoretical framework being used? What is the experience of the interviewer? Are there sample questions or a copy of the proposed script that can be included? The citation in this section refers to a questionnaire – how does the questionnaire relate to the interviewing process and the expected length of the interviews (30 minutes)?

The data analysis section could also use additional information – in particular – how is the DSAT scoring being performed? What efforts are in place to ensure reliability and validity of the scoring? How similar/different is the proposed work from other methods that have used the DSAT? How experienced are the DSAT coders for the project?

In Phase 3 methods, the authors describe how the creation of the SDM tool will be guided (by IPDAS criteria, patient input etc.) but the steps of the co-design process are not at all described. How does the co-design happen? Please include citations related to design processes that are similar to the planned work.

Based on the work described, the outcomes section seems incongruous. Including a primary outcome for each study phase does not seem consistent with the study design.

The patient and public involvement section could benefit from more details related to the foundational work that has already occurred, as it currently seems a bit vague.

The discussion is generally reasonable – but it would strengthen this section to be clearer about the unique contributions of this study in terms of the general SDM literature and the specific decision context. A clearer framing of the introduction could support tying back to this clearly in the discussion section so that the reader can see the full direction of the work.

Overall, this is an important study but the manuscript could be improved by more detail throughout as well as clearer framing in the introduction.

VERSION 1 - AUTHOR RESPONSE

Point by point responses:

Reviewer, Comment 1

"This is a protocol paper focused on a study planned to assess the acceptability of shared decision making for small cell lung cancer treatment Prophylactic cranial irradiation (PCI). This is an important choice because there are risks for complications, particularly for older adults. Based on RCTs and meta-analyses – PCI is expected to reduce the likelihood of brain metastasis – though there are recent studies which show no benefit relative to MRI screening (which would not have the same risk). In the UK, MRI is not guideline concordant treatment although a European Oncology society has recognized the need for shared decision making conversations – these are not routinely happening.
The introduction clearly explains the need for the study. The complexity of the emerging evidence contrary to RCT and other evidence needs more explanation – it isn't clear if the authors are proposing a reason for the conflicting results (could the newer studies be more representative?) or if this is conceptualized as part of the complex landscape of a tough decision for patients and their clinicians. This is particularly important given the choices the patients will be offered (PCI or no PCI each choice accompanied by standard care which does NOT include MRI screening)."

Response:
Thank you for this insightful comment. We agree that the evolving evidence base surrounding PCI required further clarification. In response, we now clarify that much of the evidence supporting PCI originates from trials conducted before the use of MRI surveillance and immunotherapy, whereas more recent studies have been undertaken in this changing diagnostic and treatment landscape. These developments may influence how benefits and risks associated with PCI are balanced and that it contributes to ongoing uncertainty regarding optimal management. Therefore, this uncertainty forms part of the complex and preference-sensitive nature of the decision faced by patients and clinicians. We have also explained further the reason why within the current UK context, MRI surveillance is not routinely available as standard care, meaning patients are typically considering PCI versus no PCI.

Reviewer, Comment 2

"The study is a single arm convergent mixed-methods design. There are three phases 1) scan for environmental evidence 2) qualitative interviews, clinic visit observations, and clinician surveys about future SDM interventions and efficacy and 3) user-centered design of an SDM intervention.
Phase 1 methods. The environmental scan is focused on evaluation of patient-facing resources for IPDAS criteria. The analytic approach seems very reasonable, but it could be argued that this is less of an environmental scan – given what may be a relatively narrow scope of materials – and more of a needs assessment focused on demonstrating the need for high quality decision aids. Although not a problem per se – it would enhance the rigor of the approach if the intention of the aim were harmonized more directly with the methods and analysis."

Response:
We appreciate this observation. This protocol describes the development of a complex intervention, which requires a needs assessment in align with the frameworks for complex interventions. Our needs assessment spans across phase 1 and 2 exploring the current information resources available to patients, current clinical practice, and experiences of patients. This needs assessment will later inform the contents of our intervention. The environmental scan is included to map the current patient-facing information provided about PCI and to assess whether these resources are adequate to support shared decision-making. Therefore, the environmental scan does form one part of the needs assessment. We understand that this might not have come across clearly. To address this concern, we have revised the manuscript to clarify that the environmental scan represents one component of a broader needs assessment informing intervention development.

Reviewer, Comment 3

"Phase 2 methods. In phase 2, the plan is to audio record consultations, collect questionnaire data, and perform semi-structured interviews. Inclusion and exclusion criteria for patients and healthcare professionals seem reasonable. The recruitment process is described as including "purposive sampling" but also employing convenience sampling. It should be made clear what the purposive sampling strategy is and how purposive sampling will be done. In addition, clarifying how convenience sampling strategies will be employed specifically in concert with purposive sampling (e.g., when purposive strategies fail? When there are insufficient numbers for purposive sampling?) should also be clarified."

Response:
We agree that the original manuscript did not clearly explain the relationship between purposive and convenience sampling within the study. We have revised the recruitment section to clarify that participant selection is guided primarily by a purposive, criterion-based sampling strategy, whereby patients, companions, and healthcare professionals are recruited based on predefined eligibility criteria. However, due to the constraints of the project and accessibility to patients, potential participants will only be recruited within a single tertiary care centre therefore incorporating elements of convenience sampling. We believe this revision provides greater transparency regarding the sampling approach and its rationale.

Reviewer, Comment 4

"The methods for data collection in phase 2 need further explication throughout. For example, in the consultation recordings section there should be more detail where will the recorder be placed in the room? What does preparing transcriptions "by the researcher" mean and how will quality be ensured?"

Response:
As part of the broader revisions to the Phase 2 methods described in response to Reviewer, Comment 3, we have expanded on the consultation recording process. The recorder will be positioned to capture clear conversations while minimising disruptions and the researcher will be in the room to ensure the recording runs smoothly. The recordings will be transcribed through Microsoft Teams transcribe function. However, to ensure the process remains researcher led, the researcher will be reviewing the audio recordings to correct any transcription errors, verify speaker identification, and capture relevant non-verbal cues or remarks that may not be accurately represented in the transcript.

Reviewer, Comment 5

"It seems that healthcare providers complete two questionnaires, and patients only the patient facing SHARED version. Is this correct? Providing some additional information about each questionnaire, and more about the content for the supplemental provider questions would improve this section of the manuscript."

Response:
We confirm that this interpretation is correct. The first questionnaire healthcare professionals will complete is the IcanSDM questionnaire before the consultation, alongside two additional statements about previous shared decision-making training (provided in the text). The second questionnaire healthcare professionals will complete is the HCP-SHARED questionnaire after the consultation. Patients complete one questionnaire after the consultation, which is the patient-facing SHARED questionnaire.

We have added extra information for each questionnaire, including what each questionnaire explores, when it is completed, which participant group completes it, and how the questionnaire is completed. We have also added the HCP-SHARED questionnaire in the Supplemental Material under appendix 1. The HCP-SHARED questionnaire was developed to support the development of this study, and its development and validation publication is in the process of submission. The SHARED questionnaire was developed to support an NHS service improvement project guided by Solberg at al's (1997) methods {Bekker, 2012, SHARED – A Patient Experience of Shared Decision Making Questionnaire} and can be accessed at (SHARED Questionnaire - Leeds Licensing).

Reviewer, Comment 6

"The qualitative interview description is quite vague. Who will complete the interviews? Have interview scripts been created? Is there a theoretical framework being used? What is the experience of the interviewer? Are there sample questions or a copy of the proposed script that can be included? The citation in this section refers to a questionnaire – how does the questionnaire relate to the interviewing process and the expected length of the interviews (30 minutes)?"

Response:
In addition to the wider revisions made to the Phase 2 methods, we have expanded the qualitative interview section to clarify that interviews will be conducted by the researcher and specify that a semi-structured interview guide will be used. We have also included that the interview guide was informed by the study aims and the wider shared decision-making and decision-support literature. We have included the interview guide in the Supplemental Material under appendix 2.

To help describe the researcher's experience and training in both qualitative and quantitative methods, we have added a separate 'Researcher Description' section in Phase 2 to explain this.

Also, thank you for highlighting the citation relating to questionnaires. The citation was included in error and has now been removed.

Reviewer, Comment 7

"The data analysis section could also use additional information – in particular – how is the DSAT scoring being performed? What efforts are in place to ensure reliability and validity of the scoring? How similar/different is the proposed work from other methods that have used the DSAT? How experienced are the DSAT coders for the project?"

Response:
To ensure the reader fully understands our use of the DSAT-10 score, we have added extra information regarding how the scoring is performed, including how each consultation will be assessed against the DSAT-10 items. We have noted the researchers prior experience and training in relation to the DSAT-10 and have explained the steps taken to support confidence, reliability and validity. This includes familiarisation with the tool, use of the published scoring guidance, repeated review of consultation recordings, and discussion with the research team. We have also explained that this method still examines decision support use and communications skills during the consultations, as the DSA-10 was originally design for. Although some other studies have used the DSAT-10 to evaluate quality decision support provided by decision coaches, this is not in scope of this study.

Reviewer, Comment 8

"In Phase 3 methods, the authors describe how the creation of the SDM tool will be guided (by IPDAS criteria, patient input etc.) but the steps of the co-design process are not at all described. How does the co-design happen? Please include citations related to design processes that are similar to the planned work."

Response:
Thank you for this comment. We agree that the co-design process was insufficiently described in the original manuscript. In response, we have revised the Phase 3 methods section to: 1) replace the term "co-development" with "co-design"; 2) outlined the planned involvement of stakeholders in providing feedback on the prototype to support iterative refinement of its content, format and usability; and 3) added references that provide the guidance and rationale for decision aid intervention development and co-design approaches, these include: *Witteman, 2021, Systematic Development of Patient Decision Aids: An Update from the IPDAS Collaboration; Witteman, 2021, An 11-Item Measure of User- and Human-Centered Design for Personal Health Tools (UCD-11): Development and Validation; and Coulter, 2013, A systematic development process for patient decision aids} An 11-item Measure of User- and Human-Centred Design for Personal Health Tools (UCD-11): Development and Validation.*

Given that this protocol describes planned research, the exact co-design activities will be defined following completion of phases 1 and 2 and further details will be provided in following publications. However, we now provide a clearer overview of the intended co-design process. Please note that implementation and evaluation of the intervention is outside the scope of the current protocol and will form part of future research.

Reviewer, Comment 9

"Based on the work described, the outcomes section seems incongruous. Including a primary outcome for each study phase does not seem consistent with the study design."

Response:
We agree that describing a primary outcome for each study phase was not consistent with the intervention development design of this study. The purpose of the individual phases is to generate evidence to inform the development and refinement of the decision support intervention, rather than to evaluate predefined outcomes. Therefore, we have removed the primary outcomes and instead described phase-specific outputs. We believe these outputs are more appropriate for the developmental nature of this research and better reflect what each phase is intended to contribute to intervention development.

Reviewer, Comment 10

"The patient and public involvement section could benefit from more details related to the foundational work that has already occurred, as it currently seems a bit vague."

Response:
Thank you for this comment. We have added more detail to the patient and public involvement section to provide greater clarity regarding the foundational PPI work already undertaken. We have explained how our patient representative assisted with the development of the study; what aspects of the protocol and participant-facing materials they commented on; how many discussions took place; and what changes this input led to. This includes clarification that their input informed the language, acceptability and relevance of the planned study materials and intervention development process.

Reviewer, Comment 11

"The discussion is generally reasonable – but it would strengthen this section to be clearer about the unique contributions of this study in terms of the general SDM literature and the specific decision context. A clearer framing of the introduction could support tying back to this clearly in the discussion section so that the reader can see the full direction of the work.
Overall, this is an important study, but the manuscript could be improved by more detail throughout as well as clearer framing in the introduction."

Response:
As we have made the introduction clearer in our argument for the work and provided clearer methodological explanation, we have altered the discussion accordingly. The revised discussion now refers not only to the study's contribution to the PCI decision context, but also to the wider SDM literature. The study contributes by expanding decision-making literature by treating SDM as a multi-level process, exploring perspectives within and beyond the clinician-patient dyad as well as adding to the limited decision-making evidence in the PCI context. We believe these revisions strengthen the Discussion and clarity of the contribution of the study.
